# Socioeconomic, demographic and healthcare determinants of the COVID-19 pandemic: an ecological study of Spain

**DOI:** 10.1186/s12889-021-10658-3

**Published:** 2021-03-29

**Authors:** Carlos Navarro García

**Affiliations:** Alfonso X University, Madrid, Spain

**Keywords:** Determinants, Spain, COVID-19, Ecological Study

## Abstract

**Background:**

The coronavirus disease 2019 (COVID-19) pandemic has posed a major challenge to health, economic and political systems around the world. Understanding the socioeconomic, demographic and health determinants affecting the pandemic is of interest to stakeholders. The purpose of this ecological study is to analyse the effect of the different socioeconomic, demographic and healthcare determinants on the mortality rate and estimated cumulative incidence of COVID-19 first wave in the Spanish regions.

**Methods:**

From the available data of the 17 Spanish regions (Autonomous Communities), we have carried out an ecological study through multivariate linear regression using ordinary least squares. To do this, we conducted an analysis using two distinct dependent variables: the logarithm of mortality rate per 1,000,000 inhabitants and the estimated cumulative incidence. The study has 12 explanatory variables.

**Results:**

After applying the backward stepwise multivariate analysis, we obtained a model with nine significant variables at different levels for mortality rate and a model with seven significant variables for estimated cumulative incidence. Among them, six variables are statistically significant and of the same sign in both models: “Nursing homes beds”, “Proportion of care homes over 100 beds”, “Log GDP per capita”, “Aeroplane passengers”, “Proportion of urban people”, and the dummy variable “Island region”.

**Conclusions:**

The different socioeconomic, demographic and healthcare determinants of each region have a significant effect on the mortality rate and estimated cumulative incidence of COVID-19 in territories where the measures initially adopted to control the pandemic have been identical.

**Supplementary Information:**

The online version contains supplementary material available at 10.1186/s12889-021-10658-3.

## Background

The emergence of the coronavirus disease 2019 (COVID-19) pandemic has created a global health emergency [1] that threatens to bring about profound social, health and economic changes in all countries.

The analysis of the effect of the different social, economic and demographic determinants on the incidence of COVID-19 is a subject of interest to researchers, as it can help to understand how the particular conditions of each geographical area have affected the spread and the impact of the disease, and can help policymakers to make decisions that can help to deal with future pandemics safely [2, 3]. Different ecological studies on the COVID-19 pandemic have shown significant associations between different socioeconomic, demographic and healthcare determinants, and the outcomes of the pandemic, both at the regional level in Europe [4–6], China [7], Iran [8], and at the country level [9, 10]. It can also be noted that the results in terms of the number of infected and dead in each country or region, as well as the effect that the pandemic is having on the economies, present enormous variations. Therefore, it is considered necessary to analyse the effect that the different social, demographic and economic determinants of an exogenous nature may have had on the effect of the pandemic in the different territories.

Spain has been one of the developed countries most affected by the first wave of the COVID-19 pandemic, with one of the highest mortality rates per inhabitant in the world during that period. The first death from COVID-19 in Spain occurred on 13 February 2020 and the country decreed a very restrictive, total lockdown on 15 March 2020, when the country already had 121 deaths. In any case, the impact of the pandemic in Spain has not been similar in all its regions, with significant differences in mortality rates and cumulative incidence depending on the geographical location. Spain has its own territorial and political characteristics that make it suitable for this analysis since, due to territorial management issues, two administrations with direct responsibilities for the management of the pandemic coexist. On the one hand, the national government is responsible for defining both public health policy and legal framework for all its territories in a comprehensive manner, so that the measures adopted to contain and mitigate the effects of the pandemic have been applied uniformly in all Spanish regions; it also corresponds to the central government to take general economic and social measures for the country as a whole. On the other hand, the organisation of the Spanish territory is characterised by the existence of different Spanish regions (called Autonomous Communities) that have a wide level of decentralisation, being responsible for, among other things, healthcare in their territories and long-term care policies. Besides, every Autonomous Community has its own social and economic determinants, which are the result of its degree of economic and social development, geographical location, natural resources and local history.

In this way, given the adoption of the same measures of containment of the effects of the pandemic for all the territories, the present ecological study allows us to deepen in the effect that the own different determinants of each territory have had on mortality and estimated cumulative incidence of the COVID-19 pandemic. The ecological study is a type of aggregate study that allows us to make inferences about the effect of different variables on the dependent variables, using groups for analysis instead of individuals, that has been shown to be effective in the analysis of similar situations.

## Methods

### Data

Administratively, Spain is divided into 17 regions (Autonomous Communities) with a high degree of decentralisation and autonomy in the management of their resources, where each is directly responsible for both the expenditure and management of the healthcare systems in its region, as well as for other aspects of economic and social nature.

This study uses two distinct dependent variables, used separately or together in studies similar to the present [4–10]: first, the mortality rate of COVID-19 per 1,000,000 inhabitants in each of the Spanish regions, calculated from the official figures of cumulative mortality rate as of 23 May 2020 in each of the regions, and provided by the Spanish Ministry of Health. The population data corresponds to 2019 and have been taken from the Spanish National Institute of Statistics. The variable is presented in logarithmic form.

The second dependent variable used is the estimated cumulative incidence, extracted from the second wave (May 2020) of the national seroprevalence studies carried out by the Spanish government [11]. This variable is expressed as a decimal. We chose this variable due to the lack of reliability of infection data confirmed by tests during the period of the study, which significantly underestimated the actual data on infected people (235,290 cumulative infections on 23 May 2020 confirmed by tests compared to the 2.44 M infected people estimated by the seroprevalence study; which is 5.2% of the total Spanish population).

As explanatory variables, 12 variables have been finally chosen, and classified into three groups: (a) healthcare and long-term care variables, (b) economic variables and (c) environmental, sociodemographic and geographical variables. The definition of each variable is given in Table 1.

**Table 1:** Explanatory Variables, descriptions and sources

Independent variables	Definition	Source
**Log public health expenditure**	Logarithm of average public health expenditure over the last 10 years (2009–2018)	Spanish Health Ministry
**Doctors**	Number of active registered doctors per 100,000 inhabitants	INE, 2018
**Hospital beds**	Number of hospital beds (public and private) per 100,000 inhabitants	Eurostat, 2017
**Nursing homes beds**	Number of places in nursing homes per 100 inhabitants over 64 years of age	Spanish Ministry of Economics, 2019
**Proportion of care homes over 100 beds**	Proportion of care homes places in care homes with more than 100 places over the total	Spanish Ministry of Economics, 2019
**Log GDP per capita**	Logarithm of GDP per capita	INE, 2019
**Aeroplane passengers**	Total number of air passenger traffic in each of the regions, in thousands	AENA, February 2020
**Proportion of elderly people**	Proportion of the population aged 65 or above, over the total.	INE, 2019
**Proportion of urban people**	Proportion of population living in municipalities with more than 100,000 inhabitants	INE, 2019
**Gini index**	Income inequality within each region	INE / EUROSTAT, 2017
**Island region**	Dummy variable indicating whether each of the regions is an island or not	
**PM2.5 concentration**	Population-weighted average of the annual average concentration of PM2.5 (μg/m3)	INE, 2018

*AENA* Spanish National Aeronautics and Air-Traffic Administration, *GDP* Gross Domestic Product, *INE* Spanish National Institute of Statistics, *PM2.5* Particulate Matter below 2.5 μm

A study of collinearity of the variables (see next section) was carried out through the variance inflation factor (VIF), from which it was concluded that there was no collinearity among the selected variables. Previously, two independent variables initially incorporated into the model were eliminated due to high collinearity: population density and research and development (R&D) expenditure per capita.

Likewise, in order to deepen the analysis of the social determinants, the dependent variable “Proportion of immigrant population in each region” was also included in the statistical analyses of both models, being in all cases not significant and not having any effect on the parameters of the two models; therefore, it has not been included in the final models to simplify their presentation.

### Statistical analysis

We have used multivariate ordinary least squares (OLS) regression model to study the relationship between each of the dependent variables and the set of explanatory variables.

The model is specified as follows:

$$ {\displaystyle \begin{array}{cc}\ln (y)={\beta}_0+{\beta}_1{\mathrm{x}}_1+{\beta}_2{\mathrm{x}}_2+{\beta}_3{\mathrm{x}}_3+\dots +{\beta}_{\mathrm{k}}{\mathrm{x}}_{\mathrm{k}}+\varepsilon & \mathrm{for}\ \mathrm{mortality}\ \mathrm{rate},\mathrm{and}\\ {}\mathrm{y}={\beta}_0+{\beta}_1{\mathrm{x}}_1+{\beta}_2{\mathrm{x}}_2+{\beta}_3{\mathrm{x}}_3+\dots +{\beta}_{\mathrm{k}}{\mathrm{x}}_{\mathrm{k}}+\varepsilon & \mathrm{for}\ \mathrm{estimated}\ \mathrm{cumulative}\ \mathrm{incidence}.\end{array}} $$

where *y* represents the dependent variable, *x*_*i*_ are the explanatory variables, *β*_*0*_ is the intercept, *β*_*i*_ are the regression coefficients and *ε* is a random component.

For the construction of the model, regressions analysis was performed through backward stepwise multiple regression analysis. Starting with the fully saturated model, the least significant independent variable was eliminated in every step, until a model with all significant variables was achieved.

An important assumption in multiple linear regressions is the independence between independent variables, so that there is no relationship between each of them, to ensure the validity of the estimation using OLS, avoiding multicollinearity problems. As indicated, in this study the evaluation of multicollinearity was performed with VIF, defined as:

$$ {VIF}_j=\frac{1}{1-{R}_j^2},j=1,2\dots, p, $$

where *p* is the number of explanatory variables, and *R*^*2*^ is the square of the coefficient of determination of the regression of *X*_*j*_ on other covariates (*p* - 1), so that:

if 0 < VIF < 5, there is no evidence of a multicollinearity problem;

if 5 < VIF < 10, there is a moderate multicollinearity problem; and.

if VIF > 10, there is a serious multicollinearity problem of variables.

The significance tests are two-sided. We consider three levels of significance for the *p*-value: < 0.1, < 0.05 and < 0.01. All statistical analyses were performed using R software.

We have used the standardised residues to perform the analysis of the assumptions of the multiple linear regression.

## Results

In this study, all the 17 Spanish regions (Autonomous Communities) have been included. The analysis of the descriptive statistics can be seen in Table 2. Descriptive analysis of non-log transformed variables can be seen in Supplementary material, Table 1.

**Table 2:** Descriptive statistics of the dependent and independent variables of all regions

Variables	N	Minimum	Maximum	Mean	SD
Log mortality per 1,000,000 inh	17	4.29	7.27	6.04	0.90
Estimated cumulative Incidence	17	0.015	0.114	0.045	0.03
Log Public Health Expenditure	17	7.03	7.40	7.24	0.09
Nursing homes beds	17	2.15	7.63	4.41	1.65
Proportion Nursing homes >100b	17	35.44	74.79	53.16	10.39
Proportion Urban population	17	8.53	71.84	36.85	14.65
Doctors per 100,000 inh	17	360.78	602.69	478.62	65.91
Hospital Beds per 100,000 inh	17	217.13	387.56	312.03	46.72
Aeroplane passengers	17	0.15	4396.90	992.13	1458.47
Island region	17	0.00	1.00	0.12	0.33
Log GDP per capita	17	9.84	10.46	10.12	0.19
PM2.5 concentration	17	7.60	15.00	10.86	1.97
Proportion > 65y population	17	0.16	0.26	0.20	0.03
Gini Index	17	0.247	0.349	0.309	.0.25

*GDP* Gross Domestic Product, *PM2.5* Particulate Matter below 2.5 μm, *SD* Standard Deviation

For the analysis of the relationship between variables, multivariate linear regressions using OLS have been carried out, using a backward stepwise approach. For the dependent variable “Log mortality per 1,000,000 inh”, the application of the selection of variables using the backward method eliminated three variables, generating the most parsimonious model with nine variables, all of them significant in different degrees. The four resulting models are summarised in Table 3. All models are statistically significant. In the final model (model 4), the VIF of the independent variables are below 5, so there are no multicollinearity problems.

**Table 3:** Backward multiple linear regression models. Dependent variable: log mortality rate

	Coefficients and variance inflation factors (VIFs)							
Explanatory variables	Model 1	VIF	Model 2	VIF	Model 3	VIF	Model 4	VIF
Constant	−39.718**		−39.699***		−41,170***		− 38,270***	
Log Public Health Expenditure	2.51	8.65	2.50	8.38	2.381	8.23	1.774*	4.27
Nursing homes beds	0.464**	4.00	0.458***	3.99	0.454***	3.70	0.430***	1.85
% Nursing homes >100b	0.017	4.10	0.016*	4.09	0.015*	3.66	0.011*	2.18
Urban population	−0.009	3.06	− 0.009	2.97	− 0.009*	2.97	− 0.010*	2.96
Doctors	−0.005**	2.85	−0.005***	2.71	−0.005***	2.40	− 0.005***	2.32
Hospital Beds	−0.004	3.75	−0.004*	3.21	−0.004*	2.70	−0.004**	2.69
Aeroplane passengers	0.0003**	7.79	0.0003**	7.40	0.0002***	5.43	0.0002***	3.95
Island region	−0.746**	2.16	−0.749***	1.79	−0.78***	1.66	−0.819***	1.46
Log GDP per capita	2.848***	5.42	2.848***	5.42.	3.00***	4.49	3.215***	2.37
PM2.5 concentration	0.033	4.09	0.033	3.98	0.029	3.90		
Gini Index	− 263.49	4.87	−263.41	4.87				
% >65y population	−0.021	2.43						
***Adjusted R***^***2***^	0.95		0.96		0.964		0.967	
***p-value***	0.003		0.000		0.000		0.000	
***Shapiro-Wilk test (p-value)***							0.45	
***Durbin-Watson test***							2.16	

*GDP* Gross Domestic Product, *PM2.5* Particulate Matter below 2.5 μm, *VIF* Variance Inflation Factor

*Significant at *p* < .1 level, **Significant at *p* < 0.05 level, and *** Significant at *p* < 0.01 level

With regard to the parameters obtained, in the three models generated, the logarithm of average public health expenditure over the last 10 years is of a positive sign and statistically significant at 10% in model 4 (1.774; *p*-value = 0.091).

Also, the variables “Number of physicians per 100,000 inhabitants” (negative sign), “Nursing home beds per 100 elderly people” (positive sign), “Aeroplane passengers” (positive sign), “Island region” (negative sign) and “Log GDP per capita” (positive sign) have statistical significance to different degrees in the three models generated.

Within model 4, the variables “Proportion of nursing homes >100 beds” and “Proportion of urban population” reach statistical significance at 10%, so that model 4 retains 9 of the 12 independent variables used, with statistical significance to different degrees.

For the dependent variable “Estimated cumulative incidence”, the backward selection of the stepwise multivariate regression analysis eliminates 5 variables, resulting in the model with 7 independent variables (model 6); all of them statistically significant in different degrees and with absence of collinearity problems. The result of the backward stepwise regression is summarised in Table 4.

**Table 4:** Backward multiple linear regression models. Dependent variable: estimated cumulative prevalence

	Coefficients and variance inflation factors (VIFs)											
Explanatory variables	Model 1	VIF	Model 2	VIF	Model 3	VIF	Model 4	VIF	Model 5	VIF	Model 6	VIF
Constant	−0.355		− 0.356		−0.427		−0.482*		−0.487*		−0.428*	
Nursing homes beds	0.018**	4.21	0.019**	4.15	0.018***	3.85	0.016***	1.94	0.016***	1.85	0.016***	1.84
% Nursing homes >100b	0.001	4.42	0.001	4.45	0.001	3.81	0.001	2.29	0.001	2.16	0.001**	1.45
Urban population	−0.0006	3.02	−0.0006	2.79	−0.0006	2.78	−0.0006	2.77	−0.0006	2.76	−0.0007*	2.34
Aeroplane passengers	0.00001	8.19	0.00001*	7.17	0.00001**	5.62	0.00001**	3.71	0.00001***	2.14	0.00001***	1.94
Island region	−0.0201	2.18	−0.0198	2.16	−0.0208	2.03	−0.0245	1.71	−0.0247*	1.71	−0.0290**	1.48
Log GDP per capita	0.025	5.58	0.026	5.49	0.031	4.08	0.047	2.20	0.050*	2.02	0.041*	1.70
% >65y population	−0.214	2.43	−0.207	2.31	−0.205	2.30	−0.223	2.23	−0.220	2.23	−0.277*	1.85
Hospital beds	−0.0001	3.56	−0.0001	3.23	−0.0001	2.89	−0.0001	2.89	−0.0001	2.47		
Log Public Health Expenditure	0.0001	8.45	0.0001	7.87	0.0001	7.80	0.00002	3.44				
PM2.5 concentration	0.002	4.09	0.002	4.07	0.002	3.90						
Gini Index	−5.426	4.94	−7.177	4.36								
Doctors	0.00002	2.90										
***Adjusted R***^***2***^	0.634		0.706		0.753		0.774		0.796		0.80	
***p-value***	0.129		0.055		0.021		0.009		0.003		0.001	
***Shapiro-Wilk test (p-value)***											0.416	
***Durbin-Watson test***											2.083	

*Significant at *p* < .1 level, **Significant at *p* < 0.05 level, and *** Significant at *p* < 0.01 level

In the aforementioned model 6, the variables “Nursing home beds per 100 elderly people” (positive sign), “Aeroplane passengers” (positive sign), “Island region” (negative sign) “Log GDP per capita” (positive sign), “Proportion of nursing homes >100 beds” (positive sign), “Proportion of urban population” (negative sign) and “Proportion of elderly people” (negative sign) are statistically significant at different levels.

## Discussion

In this ecological study, we have evaluated the impact of the different determinants at regional level that have influenced the degree of mortality rate and estimated cumulative incidence in the first wave of COVID-19 in Spain, assuming that the systems of surveillance and control of the pandemic, carried out by the national government, have been identical in all regions.

The difficulty of having individual data for analysis in this phase of the pandemic makes ecological studies a useful tool for understanding the factors that have affected the development of the pandemic. In any case, it cannot be ignored that this type of study is susceptible to bias, where the so-called ecological bias—the temptation to extrapolate the results obtained through this type of studies with aggregate data to an individual level— is the most important.

Knowing this limitation, all necessary precautions have been adopted to minimise such ecological bias, to the extent that the available data have allowed it. These precautions include the homogeneity of the analysis groups [12] by using the smallest regional unit (the Autonomous Community) for which data are available, and the mitigation of the existence of confounding factors when working with sociodemographic factors [13], especially the multicollinearity bias. In any case, the results obtained in this study should be taken with due caution, given the limitations indicated.

Having pointed this out, the present analysis carried out in Spain allows us to compare regions whose response to the initial control of the pandemic has been identical, since the measures were taken by a higher decision-making body, which allows us to isolate this aspect in the analysis of the effect of the different determinants that may have influenced the expansion and degree of affectation of the pandemic.

The statistical analysis carried out for each of the two dependent variables presents six variables with statistical significance, which are associated with both mortality and cumulative incidence.

The first finding in this study is the strong association between long-term care resources, both with mortality rate and the estimated cumulative prevalence. Thus, we can observe that the presence of a greater number of care homes is the variable most strongly associated with the mortality rate of COVID-19 in Spain, where the increase of 1 care home per 100 elderly inhabitants is significantly associated with an increase in mortality rate of 43%, keeping all other variables constant. This variable is also positively associated with a greater cumulative incidence of the disease. These findings confirm previous findings during the current pandemic [14–16]. In a similar manner, we also found a positive and statistically significant association between mortality rate and estimated cumulative incidence with the presence in the region of a greater proportion of nursing homes which size is greater than 100 places; this reinforces the idea of the relevance that the population of nursing homes has had in all the outcome figures of this pandemic in Spain.

Concerning the above, it should be noted that the proportion of the population over 64 years of age has no statistically significant effect on the mortality rate of COVID-19, showing a negative and statistically significant relationship with the estimated cumulative incidence, similar to what has been described in other regional studies, such as in the case of Germany [4] and northern Italy [6]. It seems that the effects of the pandemic in Spain have been associated not so much to the high ageing of its population as to the institutionalised elderly people. This may be explained by the fact that the non-institutionalised elderly population in Spain has followed stricter measures of self-isolation and restriction of social relations than the rest of the population, as advised by the health authorities (in Spain, during the lockdown, specific walking hours were decreed exclusively for the elderly). These measures have allowed greater control of the spread of the disease in this age group. But those institutionalised in care homes are concentrated, of advanced age and with multiple pathologies, which facilitates the spread of the virus.

Another important finding in the present study is related to the economic variables, where we find a positive and statistically significant association between the GDP per capita and both mortality and estimated cumulative incidence. Bearing in mind that the variable is presented in its logarithmic form, as is the mortality rate, we can interpret that a 1% increase in the GDP per capita is associated with a 3.21% increase in mortality rate, keeping all other variables constant. This positive association between the health outcomes of the pandemic and the income level has been observed in similar studies [10, 17]. One possible explanation is that a higher GDP per capita is an indicator of greater economic and trade dynamism, which would be indicative of greater mobility, both within and between regions, as well as with foreign countries, such that the likelihood of contagion and death would increase. In addition, we find that the number of air passengers received by each region during February 2020 (the month before the great expansion of the pandemic in Spain) presents a positive sign and is statistically significant for both dependent variables used so that an association is observed between the increased mobility of people and both incidence and mortality of the disease; this result is in agreement with other studies conducted during this pandemic [9, 18].

From the territorial and demographic point of view, it should be noted that the insularity of the territories has meant, keeping all other variables constant, a protective and statistically significant element against the estimated cumulative incidence and mortality rate of COVID-19, which seems to indicate that the geographical barriers have been, in the case of Spain, important aspects in the control of the pandemic. Likewise, our analysis shows a negative and statistically significant association between the proportion of inhabitants living in municipalities of more than 100,000 inhabitants in each region and the mortality rate of COVID-19.

The variables that consider health resources only present a significant association with mortality, not with incidence. This finding is plausible due to the fact that, in Spain, specialised care has a high weight within the Spanish public healthcare system in public health expenditure (47.47% of the total for the year 2017), which influence on the incidence of the disease is less decisive than on mortality; more decisive could be the relatively speaking, substantially lower expenditure in primary care (21.9% of the total) and public health (0.7% of the total).

Considering the above, public health expenditure, measured as the logarithm of average per capita expenditure over the last 10 years, shows a positive and statistically significant association with the mortality rate of COVID-19 in Spain. The average of the last 10 years has been chosen to incorporate into the analysis the lagging effect of public expenditure on the management of the 2020 pandemic [19]. Given that the variable is presented in its logarithmic form, as is the dependent variable, it can be interpreted as the semi-elasticity of the average public health expenditure of the last 10 years over the mortality rate, such that an increase in the average public health expenditure per capita of 1% is associated with an increase in mortality of 1.77%, all other factors remaining constant (model 4).

Although it is common to find in the literature a positive relationship between public health expenditure and the improvement of different health outcomes, such as different mortality rates or life expectancy, the evidence does not always offer conclusive results. Even in developing countries, where it is common to find evidences of a strong positive association between public health expenditure and a reduction in mortality rates and an increase in life expectancy [20, 21]. In some cases, this relationship is not so clear [22], and this is essentially related to the quality of governance in each analysed country. When the analysis is carried out in developed countries, as is our case, this relationship also appears sometimes rather blurred. In a systematic review of the topic [23], it is concluded from the analysis of 17 papers that, although the complexity of the delivery system of public healthcare services makes it difficult to demonstrate definitive associations between expenditure and outcomes, financial investments in health often have the potential to improve the health of the community.

In relation to the effect of health expenditure on the mortality rate during a pandemic, there are evidences of a significant relationship between higher health expenditure and lower mortality rates during the 2009 H1N1 influenza pandemic [24]. A study of the current pandemic finds, as does the present study, a positive and significant association between public health expenditure and mortality rate of COVID-19, in an analysis of 96 countries using Bayesian model averaging techniques [2].

There is evidence [25–27] of the existence of decreasing returns on public health expenditure in relation to health outcomes. The findings of our study seem to be compatible with the idea of decreasing returns on public health expenditure, such that, once a certain level of public health expenditure is reached, the additional gains from an additional increase of expenditure disappear, in this case in relation to the mortality rate of COVID-19.

Our study also shows a negative and statistically significant association between the number of practitioners, as well as the number of hospital beds, and the mortality rate of COVID-19, results that confirm previous findings within the present pandemic outcomes [28]; in the case of practitioners, an increase of 1 unit in the ratio of practitioners per 100,000 inhabitants is associated with a reduction in the mortality rate of COVID-19 by 0.5%. In the case of hospital beds, an increase of 1 unit in the ratio of beds per 100,000 inhabitants reduces the mortality rate of COVID-19 by 0.3%, all other factors remaining constant. It should be kept in mind that in this study both variables represent the total of both resources in both the public and private health sectors, the latter representing approximately 30% of total Spanish healthcare, measured in terms of expenditure.

With regard to environmental aspects, our study does not find a significant relationship between PM2.5 concentration levels and increased mortality rate of COVID-19, as has been shown by other studies of this pandemic [29, 30].

Our study does not either find significant associations between the variables of social structure and inequity, and the outcomes of the pandemic.

## Conclusions

The ecological analysis carried out in the present study indicates that the effect of the estimated cumulative incidence and mortality rate of COVID-19 in the different regions of the Spanish state is notably influenced by the social, economic, healthcare and demographic characteristics of each region. In particular, after the analysis of two distinct dependent variables for the same group of independent variables, we found a total of six variables with statistical significance and of the same sign in both models, which leads us to greater robustness of the results obtained in the present study. The detailed analysis of these determinants can help policymakers to make decisions to mitigate the effects of future hypothetic pandemic scenarios.

## Supplementary Information

**Additional file 1:** **Table 1.** Descriptive analysis of non-log transformed variables.

## Data Availability

The datasets supporting the conclusions of this article are public and are available in the repositories listed in Table 1.

## Abbreviations

AENA: Spanish National Aeronautics and Air-Traffic Administration
GDP: Gross Domestic Product
INE: Spanish National Institute of Statistics
PM2.5: Particulate Matter below 2.5 μm
SD: Standard Deviation
VIF: Variance Inflation Factor
OLS: Ordinary Least Squares
R&D: Research and Development
