# Supplementary material for: Socioeconomic, demographic and healthcare determinants of the COVID-19 pandemic: an ecological study of Spain
Source: BMC Public Health. 2021 Mar 29;21:606. doi: 10.1186/s12889-021-10658-3 (PMC8006121; doi:10.1186/s12889-021-10658-3)
Supplement: Supplementary file 1 — Additional file 1: Table 1. Descriptive analysis of non-log transformed variables. [file 12889_2021_10658_MOESM1_ESM.docx]

**Supplementary material**

**Table 1. Descriptive analysis of non-log transformed variables**

| **Variables** | **N** | **Minimum** | **Maximum** | **Mean** | **SD** |
| --- | --- | --- | --- | --- | --- |
| Mortality x 1,000,000h. | 17 | 72.90 | 1443.28 | 580.82 | 431.94 |
| Public heath expenditure | 17 | 1134.01 | 1643.67 | 1405.81 | 129.65 |
| GDP per capita | 17 | 18769 | 35041 | 25277.82 | 5016.29 |
